# Improvement of the physical properties of ZnO/CdTe core-shell nanowire arrays by CdCl_2_ heat treatment for solar cells

**DOI:** 10.1186/1556-276X-9-222

**Published:** 2014-05-07

**Authors:** Vincent Consonni, Sébastien Renet, Jérôme Garnier, Patrice Gergaud, Lluis Artús, Jérôme Michallon, Laetitia Rapenne, Estelle Appert, Anne Kaminski-Cachopo

**Affiliations:** 1 Univ. Grenoble Alpes, LMGP, Grenoble F-38000, France; 2 CNRS, LMGP, Grenoble F-38000, France; 3 CEA, LETI, MINATEC Campus, Grenoble F-38054, France; 4 Institut Jaume Almera, Consell Superior d'Investigacions Científiques (CSIC), Lluís Solé i Sabarís s.n, Barcelona Catalonia 08028, Spain; 5 Univ. Grenoble Alpes, IMEP-LAHC, Grenoble F-38000, France; 6 CNRS, IMEP-LAHC, Grenoble F-38000, France

**Keywords:** ZnO/CdTe, Nanowire arrays, CdCl_2_, Heat treatment, Solar cells

## Abstract

CdTe is an important compound semiconductor for solar cells, and its use in nanowire-based heterostructures may become a critical requirement, owing to the potential scarcity of tellurium. The effects of the CdCl_2_ heat treatment are investigated on the physical properties of vertically aligned ZnO/CdTe core-shell nanowire arrays grown by combining chemical bath deposition with close space sublimation. It is found that recrystallization phenomena are induced by the CdCl_2_ heat treatment in the CdTe shell composed of nanograins: its crystallinity is improved while grain growth and texture randomization occur. The presence of a tellurium crystalline phase that may decorate grain boundaries is also revealed. The CdCl_2_ heat treatment further favors the chlorine doping of the CdTe shell with the formation of chlorine A-centers and can result in the passivation of grain boundaries. The absorption properties of ZnO/CdTe core-shell nanowire arrays are highly efficient, and more than 80% of the incident light can be absorbed in the spectral range of the solar irradiance. The resulting photovoltaic properties of solar cells made from ZnO/CdTe core-shell nanowire arrays covered with CuSCN/Au back-side contact are also improved after the CdCl_2_ heat treatment. However, recombination and trap phenomena are expected to operate, and the collection of the holes that are mainly photo-generated in the CdTe shell from the CuSCN/Au back-side contact is presumably identified as the main critical point in these solar cells.

## Background

Increasing interest has been devoted to core-shell semiconductor nanowires (NWs) over the past years due to their potential use in energy-harvesting devices such as nanostructured solar cells [1,2]. Semiconductor NWs are expected to offer an efficient charge carrier transport and collection, thanks to their very high crystalline quality [2]. The core-shell NW heterostructure can also benefit from the charge carrier separation over a small distance of the NW diameter [2]. Furthermore, the NW arrays can act as a photonic crystal, which in turn improves significantly light absorption and trapping [2].

Owing to its wide bandgap energy of 3.3 eV at room temperature, high exciton binding energy of 60 meV, and high electron mobility, increasing efforts have been dedicated to the development of ZnO nanostructures [3,4]. The ability of ZnO to grow as NWs by a wide variety of chemical deposition techniques such as metalorganic or standard chemical vapor deposition [5,6], electrodeposition [7], and chemical bath deposition (CBD) [8,9] is very attractive. ZnO NWs have therefore emerged as promising building blocks for nanostructured solar cells such as dye- and quantum dot-sensitized solar cells as well as extremely thin absorber solar cells, all of them including the type-II band alignment [10-13]. The latter offer an alternative route to the conventional p-n junction that suffers from the doping difficulty in some of the compound semiconductors belonging to the III-V or II-VI groups [14]. The type-II band alignment occurs when one of the two semiconductors in the core-shell structure has the energy minimum of both the conduction and valence bands [15]. The alignment is expected to induce an efficient charge carrier separation as well as an alternative absorption channel *via* the type-II optical transition [13,15], which may significantly improve the light absorption and efficiency of nanostructured solar cells.

Owing to its bandgap energy of 1.5 eV at room temperature and its high optical absorption coefficient (>10^4^ cm^-1^), CdTe is a very efficient absorbing layer and considered as a good candidate as the shell layer. The potential scarcity of tellurium should also be emphasized and may require the forthcoming use of CdTe in nanostructures in order to reduce the amount of raw materials consumed. In particular, solar cells made from ZnO/CdTe planar structures grown by spray pyrolysis or solution process have reached the photo-conversion efficiency of 8.8% and 12.3%, respectively, which clearly indicates their promising potential photovoltaic applications [16-18]. ZnO/CdTe nanocone tip/film structures have lead to the fabrication of solar cells with a photo-conversion efficiency as high as 3.2% [19]. The development of ZnO/CdTe core-shell NW arrays grown by a wide variety of low-cost deposition techniques has therefore been attracting much attention [20-33]. This is supported by the systematic optical simulations of their ideal short-circuit current density, showing that the absorption capability is highly favorable in ZnO/CdTe core-shell NW arrays and even better than in Si core-shell NW arrays [20]. Levy-Clément *et al.* have first deposited ZnO/CdTe core-shell NW arrays by using electrodeposition and vapor phase epitaxy, respectively [21]. In the radial structure, the CdTe shell composed of nanograins (NGs) can be grown on ZnO NWs by vapor-phase epitaxy [21], MOCVD [22], electron beam deposition [23,25,28], electrodeposition [27,33], close space sublimation [30] or successive ion layer adsorption and reaction (SILAR) [31]. An alternative route is to deposit CdTe nanoparticles (NPs) on ZnO NWs by immersion or dip coating [24,26,29,32]. In both routes, a uniform deposition of the CdTe shell has been reported from the bottom to the top of ZnO NWs. Still, the photovoltaic properties of the resulting nanostructured solar cells are fairly poor [22,24,25,27,29,32]. One explanation may be correlated to the thermal activation of CdTe NGs and NPs. For instance, it is well-known for p-CdTe/n-CdS heterojunctions that the use of CdCl_2_ heat treatment can significantly enhance the photovoltaic properties of the resulting solar cells [34]. The CdCl_2_ heat treatment is expected to favor recrystallization of grains [34-37] as well as passivation of grain boundaries (GBs) [38]; these are beneficial for the transport properties of the resulting solar cells [39]. Nevertheless, very little is known concerning the effects of the CdCl_2_ heat treatment on the physical properties of ZnO/CdTe core-shell NW arrays. It is the aim of this paper to reveal the chemical and physical mechanisms following the CdCl_2_ heat treatment in ZnO/CdTe core-shell NW arrays as well as their effects on the photovoltaic performances.

## Methods

### Synthesis of ZnO/CdTe core-shell NW arrays on FTO thin films

The synthesis of ZnO/CdTe core-shell NW arrays was achieved on fluorine-doped tin oxide (FTO) thin films by using low-cost chemical and physical deposition techniques. Polycrystalline FTO thin films were initially deposited by ultrasonic spray pyrolysis on a Corning C1737 borosilicate glass substrate (Delta Technologies, Ltd., CO, USA) heated at a growth temperature of 420°C. The chemical precursor solution was composed of 0.16 M of SnCl_4_ · 5H_2_O and 0.04 M of NH_4_F in a methanolic solution and sprayed at a constant flow rate of 1.25 mL/min for a given volume of 20 mL. The thickness of the FTO thin films is about 300 nm. The growth texture of the FTO thin films was controlled along the <100 > orientation in order to favor the structural ordering of the layers grown on top of them [40,41]. The optical transmittance and electrical resistivity of the FTO thin films are about 90% and a few 10^-4^ Ω · cm, respectively. A seed layer of ZnO NPs was then grown at room temperature by dip coating. The chemical precursor solution consisted of zinc acetate dihydrate (ZnAc_2_·2H_2_O) and monoethanolamine dissolved in absolute ethanol in an equimolar ratio of 0.375 M. The withdrawal speed of 3.3 mm/s was used. All of the samples were initially pre-heated on a hot plate kept at 300°C for 10 min and subsequently post-heated on another plate at 540°C for 1 h. The thickness of the seed layer is about 20 nm. The growth texture of the seed layer was induced along the *c*-axis in order to favor the vertical alignment of ZnO NWs grown on top of them [42,43]. Subsequently, the ZnO NWs were grown by CBD for 3 h in a chemical precursor solution of zinc nitrate hexahydrate (Zn(NO_3_)_2_·6H_2_O) and hexamethylenetetramine (C_6_H_12_N_4_) mixed in an equimolar ratio of 0.025 M, dissolved in de-ionized water, and heated at 90°C. CdTe NGs were eventually deposited for 6 min by close space sublimation from a source of CdTe 5 N powder heated at 480°C. The ZnO/CdTe core-shell NW arrays were dipped in a saturated CdCl_2_:methanol solution for 30 min and then annealed under argon atmosphere for 1 h at different annealing temperatures in the range of 300°C to 500°C.

### FESEM, XRD, Raman scattering, PL, and absorption measurements

The structural properties of the ZnO/CdTe core-shell NW arrays were investigated by field-emission scanning electron microscopy (FESEM), high-resolution transmission electron microscopy (HRTEM), X-ray diffraction (XRD) measurements, and Raman scattering measurements. FESEM images were recorded with a ZEISS Ultra 55 microscope (Oberkochen, Germany). HRTEM specimens were prepared by dispersing ZnO/CdTe core-shell NWs kept in an ethanol solution on a copper grid. HRTEM images were recorded with a JEOL JEM-2010 microscope (Tokyo, Japan) operating at 200 kV. XRD patterns were collected with a PanAlytical diffractometer (Almelo, The Netherlands) using CuKα radiation according to the Bragg-Brentano configuration. The texture of the CdTe shell was quantitatively analyzed from the K_α1_ component in the framework of the Harris method by determining both the degree of preferred orientation and texture coefficients [40,41]. The *θ*-2*θ* XRD measurements were performed in the range of 20° to 100° (in 2*θ* scale). Seven CdTe diffraction peaks were taken into account for the texture analysis: (111), (220), (311), (400), (331), (422), and (531). The (511) diffraction peak was excluded from the texture analysis, as being superimposed with the (333) diffraction peak. The intensity of each CdTe diffraction peak was precisely determined by pseudo-Voigt fits, and their deconvolution with other SnO_2_ or ZnO diffraction peaks was carefully achieved when required. The 00-041-1445, 00-036-1451, and 00-0150770 files of the International Center for Diffraction Data (ICDD) were used for SnO_2_, ZnO, and CdTe, respectively. Absorption measurements were performed with a UV-visible-NIR Perkin Elmer Lambda 950 spectrophotometer (Waltham, MA, USA). An integrating sphere was used for light-harvesting efficiency measurements by determining the total optical transmittance and reflectance. The 5 K PL measurements were achieved in a helium flow cryostat by using a frequency-doubled argon laser operating at 244 nm. The 5 K PL spectra were analyzed by using a spectrometer equipped with a 600-line/mm grating and detected with a liquid-nitrogen cooled charge-coupled device (i.e., CCD detector). The excitation power was varied by using an optical attenuator. For all of the PL spectra, the spot size was about 100 μm. Raman measurements were performed with an argon laser operating at 514.5 nm, and the scattered light was analyzed using a Jobin-Yvon T64000 triple spectrometer (Palaiseau, France) equipped with a CCD detector. Raman spectra were collected in the frequency range of 80 to 700 cm^-1^ at room temperature in near-backscattering geometry using the subtractive configuration of the spectrometer with 100-μm slits (spectral width ≈ 2.2 cm^-1^). For all of the Raman spectra, the excitation power and spot size were about 2.5 mW and 1 μm, respectively. In order to investigate the homogeneity of the ZnO/CdTe core-shell NW arrays at micron and submicron scales, a Marzhauser Wetzlar motorized stage (Wetzlar, Germany) was used with a lateral step resolution of 100 nm either in steps of 200 nm or 3 μm.

### Solar cell fabrication and photovoltaic performances

In order to investigate the photovoltaic properties of as-grown and annealed ZnO/CdTe core-shell NW arrays, CuSCN as a wide bandgap p-type semiconductor was deposited by impregnation. A saturated solution of CuSCN was initially prepared by dissolving 50 mg of CuSCN in 10 mL of n-propyl sulfide. The solution of 0.04 M was then spread over the ZnO/CdTe core-shell NW arrays held on a hot plate kept at 100°C. The solar cells were completed by evaporating a 40-nm-thick gold contact with an Edwards evaporator (Gennevilliers, France). Their photovoltaic properties were recorded under 100 mW/cm^2^ AM 1.5G simulated sunlight (model 96000, Oriel Instruments, Irvine, CA, USA). The solar simulator had previously been calibrated by using a NREL certified solar cell (Spectra Nova, Ontario, Canada). The external quantum efficiency (EQE) measurements were achieved by using a halogen lamp as the light source and a Newport monochromator (Cornestone 130, Irvine, CA, USA). The acquisition was collected *via* a lock-in amplifier system. A silicon calibrated diode was used for determining the absolute incident-light intensity.

In order to analyze the spatial distribution of photo-generated charge carriers, the optical generation rate was computed with a three-dimensional (3D) rigorous coupled wave analysis (RCWA) tool developed at IMEP-LAHC [44]. The optical generation rate basically represents the number of photo-generated charge carriers per unit volume and unit time. The 3D monochromatic generation rate was calculated for each wavelength (*λ*), ranging from *λ* = 300 nm to *λ* = 820 nm with a *λ* step of 20 nm, from:

(1) $$G\left(r,\theta ,z,\lambda \right)=\frac{\pi \cdot ℑ\left[\varepsilon \left(r,\theta ,z,\lambda \right)\right]\cdot {\left|E\left(r,\theta ,z,\lambda \right)\right|}^{2}}{h},$$

where *λ*, *E*, and *h* are the permittivity, electric field amplitude, and Planck constant, respectively. *r*, *θ*, and *z* are the variables of the cylindrical coordinate system used. The optical databases were taken from [20,45,46], G Rey *et al*., unpublished work] for ZnO, CdTe, CuSCN, and FTO, respectively. The 3D monochromatic generation rate was averaged over a circle perimeter following the procedure of [47,48].

(2) $$G\left(r,z,\lambda \right)=\frac{1}{2\pi }\int_{\theta =0}^{2\pi }G\left(r,\theta ,z,\lambda \right)d\theta$$

Eventually, the 3D polychromatic generation rate was computed by weighting the 3D monochromatic generation rates with the solar irradiance spectrum (*I*_AM1.5G_ taken from [49]):

(3) $$G\left(r,z\right)=\int_{\lambda }\frac{I_{\text{AM}1.5g}\left(\lambda \right)}{I_{i\text{ncident}}}G\left(r,z,\lambda \right)d\lambda ,$$

where *I*_incident_ is the light intensity shining the ZnO/CdTe core-shell NW arrays from the FTO/glass substrate side.

## Results and discussion

### Effects on the structural ordering of ZnO/CdTe core-shell NW arrays

**Figure 1:**
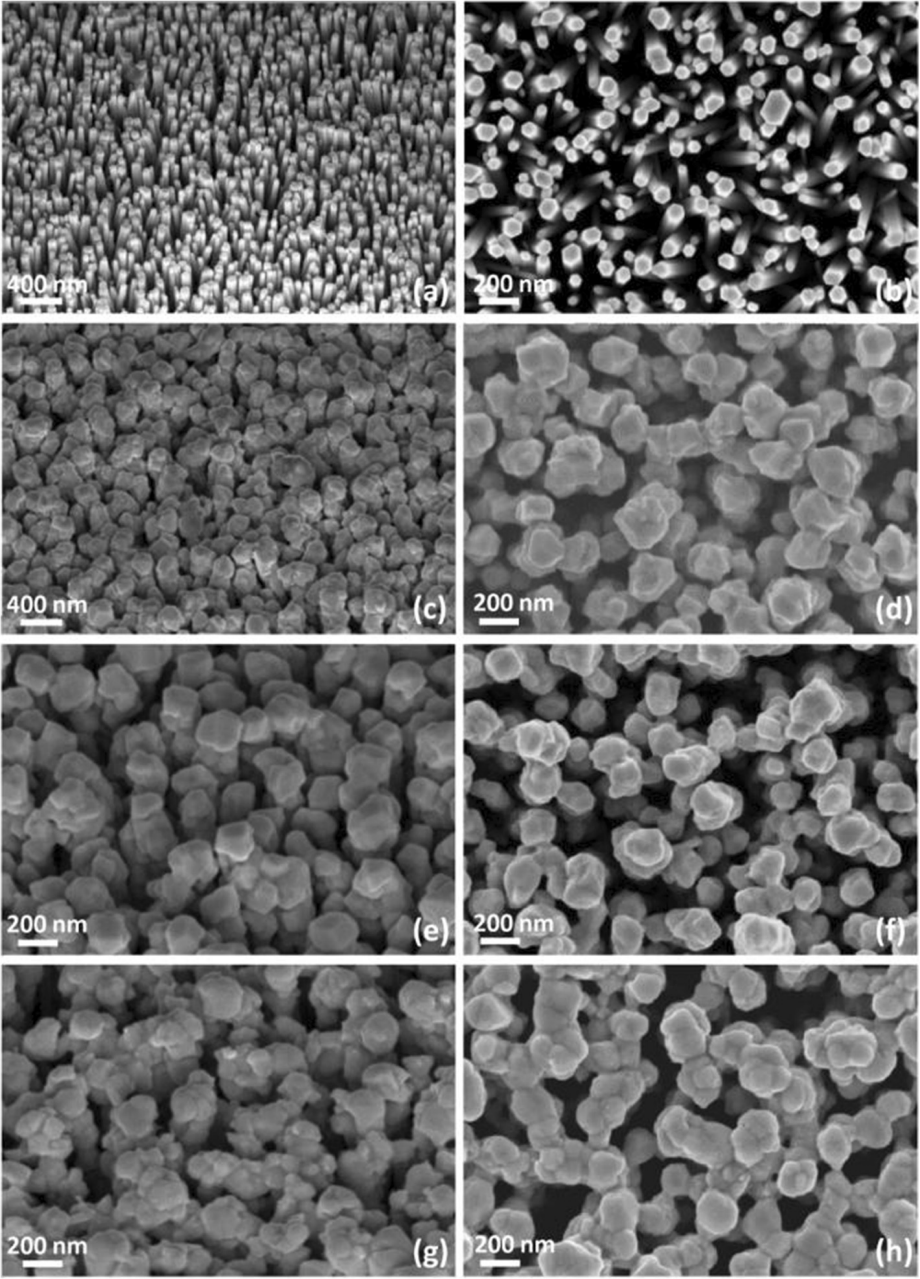
**FESEM images. (a, c, e, g)** 40° tilted view and **(b, d, f, h)** top view of the **(a, b)** as-grown bare ZnO NWs, **(c, d)** as-grown ZnO/CdTe core-shell NW arrays, and ZnO/CdTe core-shell NW arrays annealed at **(e, f)** 300°C and **(g, h)** 450°C for 1 h, respectively.

**Figure 2:**
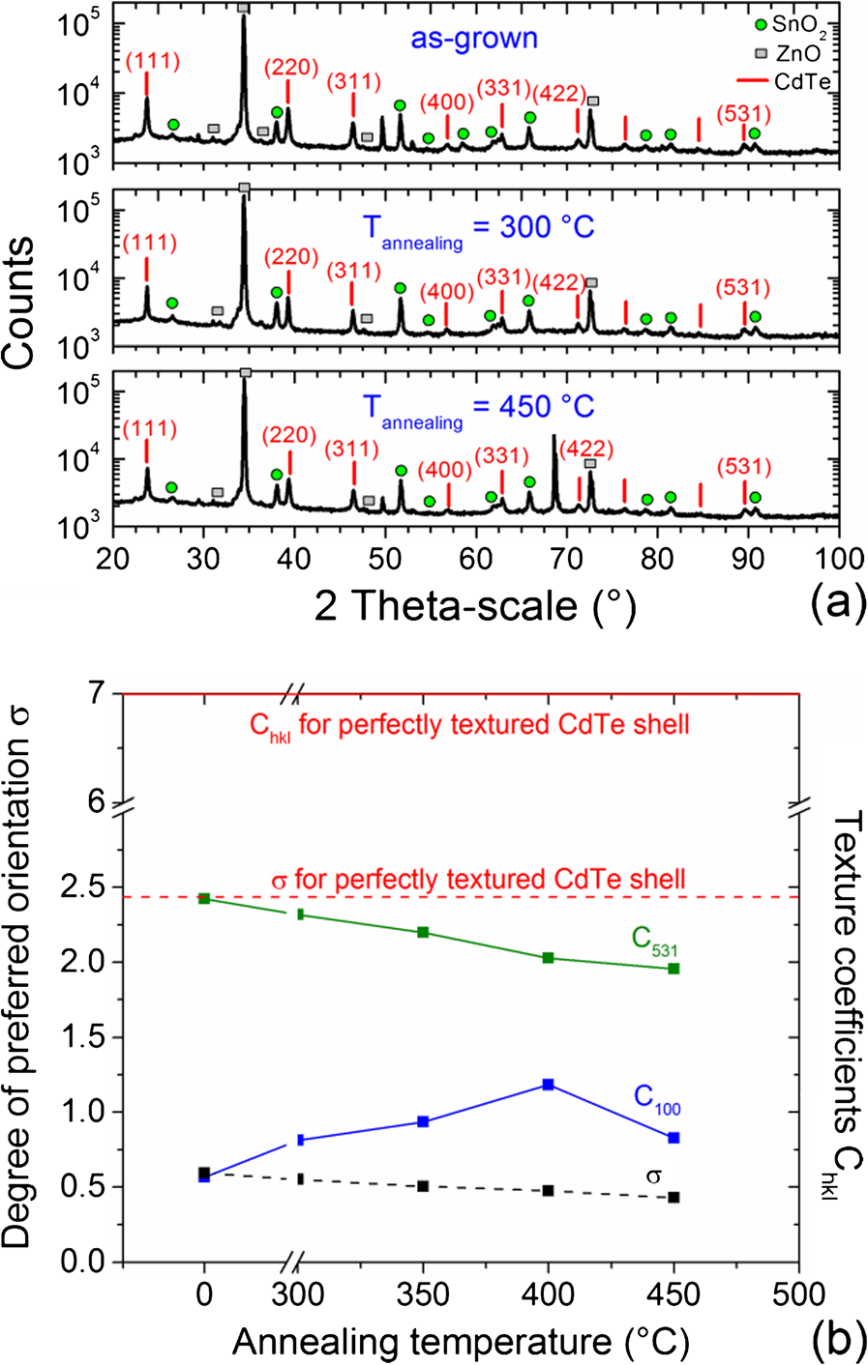
**XRD patterns, degree of preferred orientation, and texture coefficients. (a)** XRD patterns of the as-grown and annealed ZnO/CdTe core-shell NW arrays at 300°C and 450°C for 1 h. **(b)** Degree of preferred orientation as well as <531 > and <100 > texture coefficients C_531_ and C_100_ as a function of annealing temperature.

**Figure 3:**
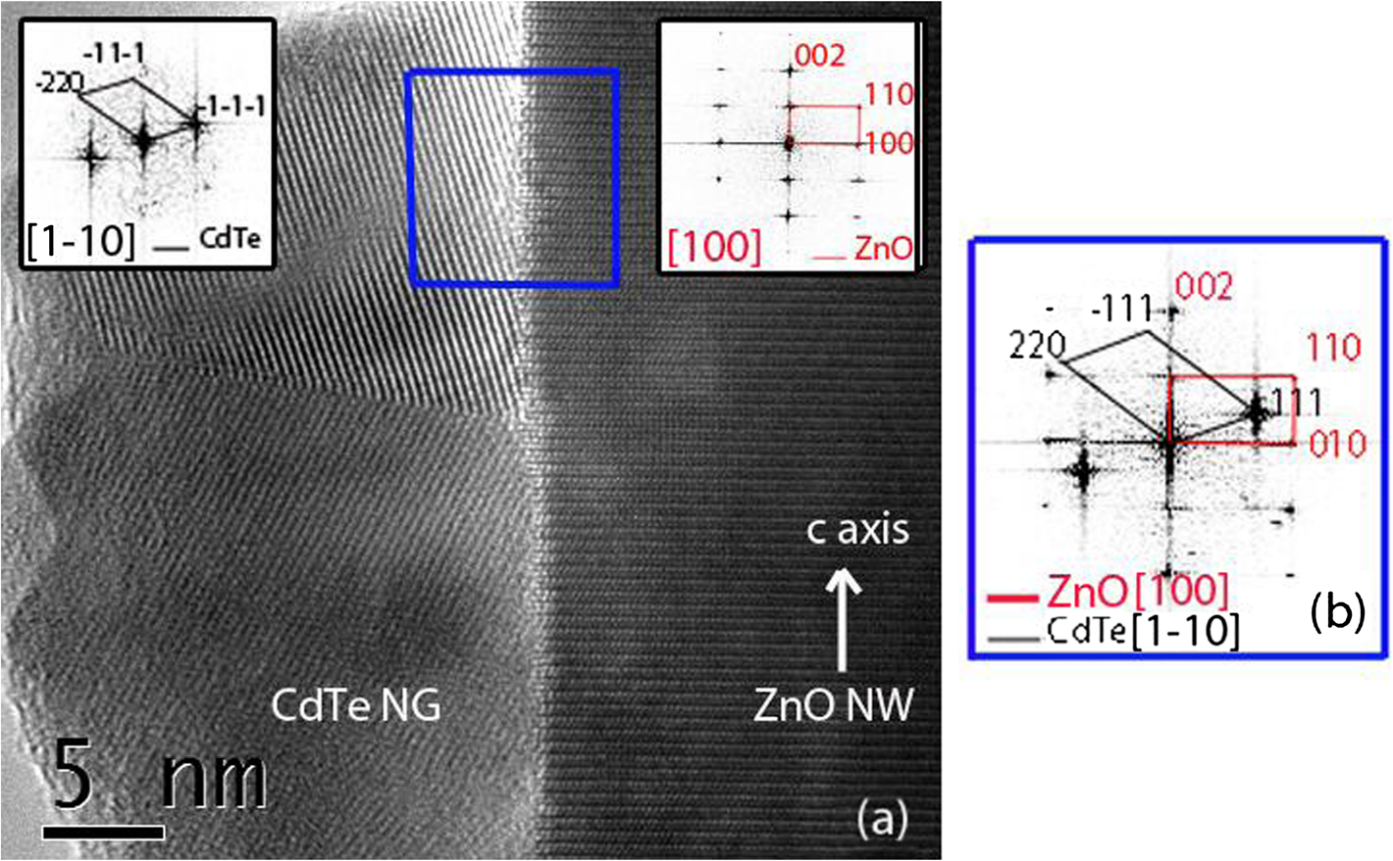
**HRTEM image and Fourier-filtered enhancement. (a)** HRTEM image of an as-grown ZnO/CdTe core-shell NW. The insets are Fourier-filtered enhancements along the [100] and [1-10] zone axes of the ZnO NW and CdTe NG, respectively. **(b)** Fourier-filtered enhancement collected at the ZnO/CdTe interface, as depicted in the blue rectangular area in **(a)**.

The structural properties of the as-grown and annealed ZnO/CdTe core-shell NW arrays are presented in Figures 1, 2 and 3. ZnO NWs present a mean diameter and length of about 50 to 100 nm and 1 μm, respectively. They have a wurtzite structure and are *c*-axis-oriented on the seeded FTO thin films, as shown in Figures 2 to 3. Interestingly, the ZnO NWs homoepitaxially form on the seed layer, especially on the free surface of polar *c*-plane ZnO NPs [42,43]. Their growth is limited by the mass transport of chemical precursors in solution. Both the structural morphology of the ZnO seed layer and the chemicals used in solution govern the typical structural properties of the ZnO NWs such as their length, diameter, and density. The ZnO NWs are not perfectly aligned vertically (i.e., slightly tilted with respect to the normal to the surface) since the polar *c*-plane ZnO NPs exhibit a significant mosaicity (i.e., the *c*-plane is slightly tilted with respect to the surface plane). Furthermore, ZnO NWs are twisted to each other since the seed layer does not have any in-plane orientation [50], as expected in polycrystalline thin films, and hence drives their in-plane orientation by homoepitaxial relationship.

Importantly, the CdTe NGs uniformly cover the ZnO NWs from their bottom to their top both for as-grown and annealed ZnO/CdTe core-shell NW arrays. The CdTe shell thickness varies in the range of 50 to 100 nm and is typically larger on top of the ZnO NWs than on the vertical sidewalls. This indicates that a larger amount of CdTe is deposited on top of the ZnO NWs. The crystallite size as deduced from the Debye-Scherrer law is instead about 32 nm and thus is smaller than the range of the CdTe shell thickness, showing that several layers of CdTe NGs have been deposited. Basically, it also turns out that some CdTe NGs can cover several ZnO NWs, as depicted in Figure 1. The as-grown CdTe NGs have a zinc-blend structure and are polycrystalline, as shown by the XRD patterns in Figure 2a. No epitaxial relationships are thus expected with ZnO NWs since no strong preferential orientation is revealed. This is further shown on the local scale by HRTEM imaging and Fourier-filtered enhancements in Figure 3. No sign of the presence of a transitional layer is further revealed in Figure 3, which excludes the formation of ternary compounds, for instance, in agreement with the XRD patterns of Figure 2a. The absence of epitaxial relationship is likely due to (i) the very high lattice mismatch between ZnO and CdTe and to (ii) the high growth rate for the deposition of CdTe by CSS that typically lies in the range of 0.5 to 1 μm/h. This is also usual for the deposition of CdTe by CSS in the form of thin films. In contrast, some epitaxial relationships have been reported for ZnO/ZnSe core-shell NW arrays, despite the polycrystalline nature of the ZnSe shell [13]; however, the growth rate for the deposition of the ZnSe shell by pulsed laser deposition is instead much lower and of the order of 0.03 μm/h, favoring the establishment of epitaxial relationships. The growth of CdTe NGs by CSS basically follows the Volmer-Weber mechanisms [30]: 3D islands initially nucleate on the vertical sidewalls and top of the ZnO NWs, then coarsen, and eventually coalesce to form a continuous 2D shell. Interestingly, the CdTe NGs are preferentially oriented along the <531 > direction: the degree of preferred orientation as deduced from the Harris method is 0.6, corresponding to a <531 > texture coefficient of 2.4, as shown in Figure 2b. The texture magnitude is hence not pronounced, as expected for polycrystalline thin films deposited by CSS in contrast to standard physical vapor deposition or sputtering [51]. The texture of CdTe NGs can be accounted for by thermodynamic considerations (as usually achieved for polycrystalline thin films), for which grain growth is driven by the minimization of total free energy. The total free energy is dependent upon surface, interface, and strain energy, which are strongly anisotropic in CdTe (i.e., the anisotropy factor is equal to 2.32) [52]. Here, CdTe NGs have yielded (the yield stress being fairly low), and the strain is plastically accommodated; Σ3 deformation twins, and dislocations are formed. The stored strain energy within a grain is however expected to be insufficient for further relaxation in nearby grains: accordingly, the strain energy depends on both the yield stress and elastic biaxial modulus. The <531 > texture is thus governed by strain energy minimization since the <531 > direction has one of the lowest biaxial elastic modulus [53]. The growth of the as-grown CdTe NGs on ZnO NWs preserves the typical growth regimes for their planar growth. However, the critical film thickness separating the growth regimes driven by surface or strain energy minimization is strongly decreased.

Upon the CdCl_2_ heat treatment of the ZnO/CdTe core-shell NW arrays, CdTe NGs significantly grow and their crystallization is enhanced; the formation of the well-defined facets and GBs is shown in Figure 1 for high annealing temperature. Also, their crystallite size is increased up to 56 nm as annealing temperature is raised to 400°C. For higher annealing temperature, the crystallite size decreases with film thickness, owing to CdTe sublimation. The growth of CdTe NGs upon annealing is driven by diffusion-induced GB migration, which is assisted by impurity atoms [54,55]. Interestingly, the texture of the annealed CdTe NGs along the <531 > direction is decreased, corresponding to randomization phenomena [35-37,51,56]. The degree of preferred orientation and <531 > texture coefficient decrease down to 0.4 and 1.9, respectively, as annealing temperature is raised to 450°C, as revealed in Figure 2b. The slight deterioration of the <531 > texture of CdTe NGs on ZnO NWs after CdCl_2_ heat treatment can be compared with the slight deterioration of the <111 > texture of polycrystalline CdTe thin films above a threshold annealing temperature [37,56]. In contrast, the texture of the annealed CdTe NGs is strengthened along the <100 > direction as annealing temperature is raised to 400°C. The <100 > texture is governed by strain energy minimization [52,53]. The underlying physical process upon CdCl_2_ heat treatment is still unclear, but it has recently been suggested that the formation of CdTe-CdCl_2_ eutectic liquid phases at GBs may favor recrystallization phenomena through the generation of compressive stresses [56].

**Figure 4:**
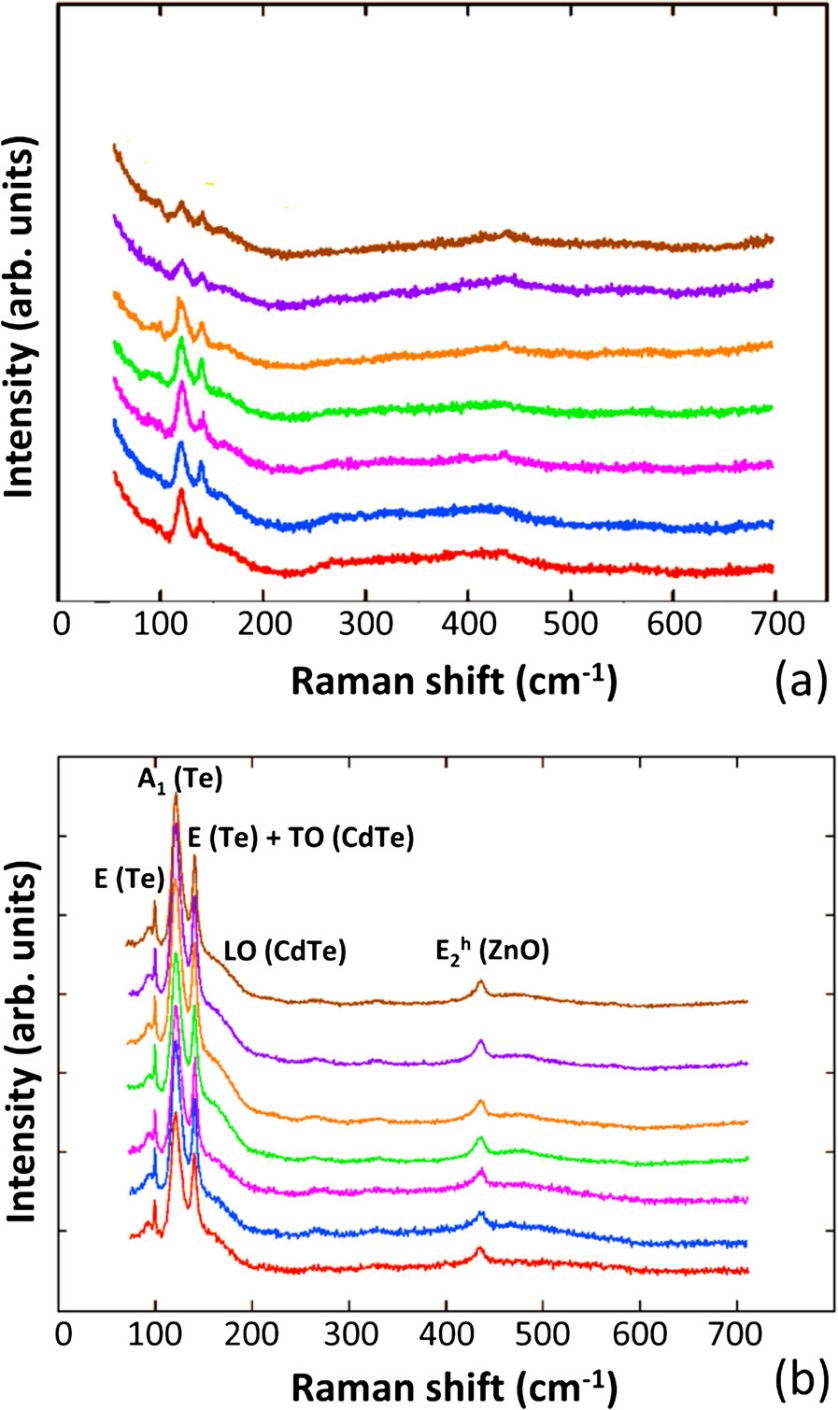
**Raman scattering measurements.** Room-temperature Raman measurements of **(a)** as-grown and **(b)** annealed ZnO/CdTe core-shell NW arrays at 450°C for 1 h obtained by laterally moving the stage each 200 nm. The Raman spectra collected by moving the stage each 3 μm are identical. The excitation power and beam size are 2.5 mW and 1 μm, respectively.

The Raman spectra of the as-grown and annealed ZnO/CdTe core-shell NW arrays are presented in Figure 4. For all of the spectra, a Raman peak points at 438 cm^-1^, corresponding to the $E_{2}^{h}$ mode of ZnO [57]. A wide number of Raman peaks related to CdTe arises in the frequency range below 200 cm^-1^. In particular, three sharp peaks at 92, 121, and 140 cm^-1^ and a shoulder at about 158 cm^-1^ are revealed in the low-frequency range. Importantly, the presence of a tellurium crystalline phase has previously been shown by Raman scattering in CdTe crystals: the Raman peaks at 92 and 121 cm^-1^ correspond to the E and A_1_ phonon modes of crystalline tellurium, respectively [58]. Also, the peak at 140 cm^-1^ can be assigned to a superposition of the E mode of crystalline tellurium and of the transverse optical (TO) mode of CdTe. The shoulder observed in the Raman spectra around 158 cm^-1^ can more likely be associated with the longitudinal optical (LO) modes of CdTe, which have been found at about 168 cm^-1^ in [58]. Since the tellurium precipitates can decorate GBs, the occurrence of a tellurium crystalline phase in as-grown and annealed ZnO/CdTe core-shell NW arrays may be related to the high density of GBs in CdTe NGs. By further comparing both Raman spectra, it turns out that the crystallinity is strongly improved after CdCl_2_ heat treatment. This reveals that the ZnO/CdTe core-shell NW arrays undergo recrystallization phenomena upon CdCl_2_ heat treatment, in agreement with FESEM images and XRD measurements. Furthermore, the intensity of the Raman peak at 438 cm^-1^ corresponding to the ZnO NWs is slightly increased after the CdCl_2_ heat treatment. This suggests that the CdCl_2_ heat treatment results in a slight decrease in the thickness of CdTe NGs in some regions. This is also shown by absorption measurements, in which the total optical transmittance is increased after CdCl_2_ heat treatment as annealing temperature is raised from 300°C to 450°C. Eventually, CdTe NGs are completely sublimated at an annealing temperature of 500°C.

### Effects on the doping properties of ZnO/CdTe core-shell NW arrays

**Figure 5:**
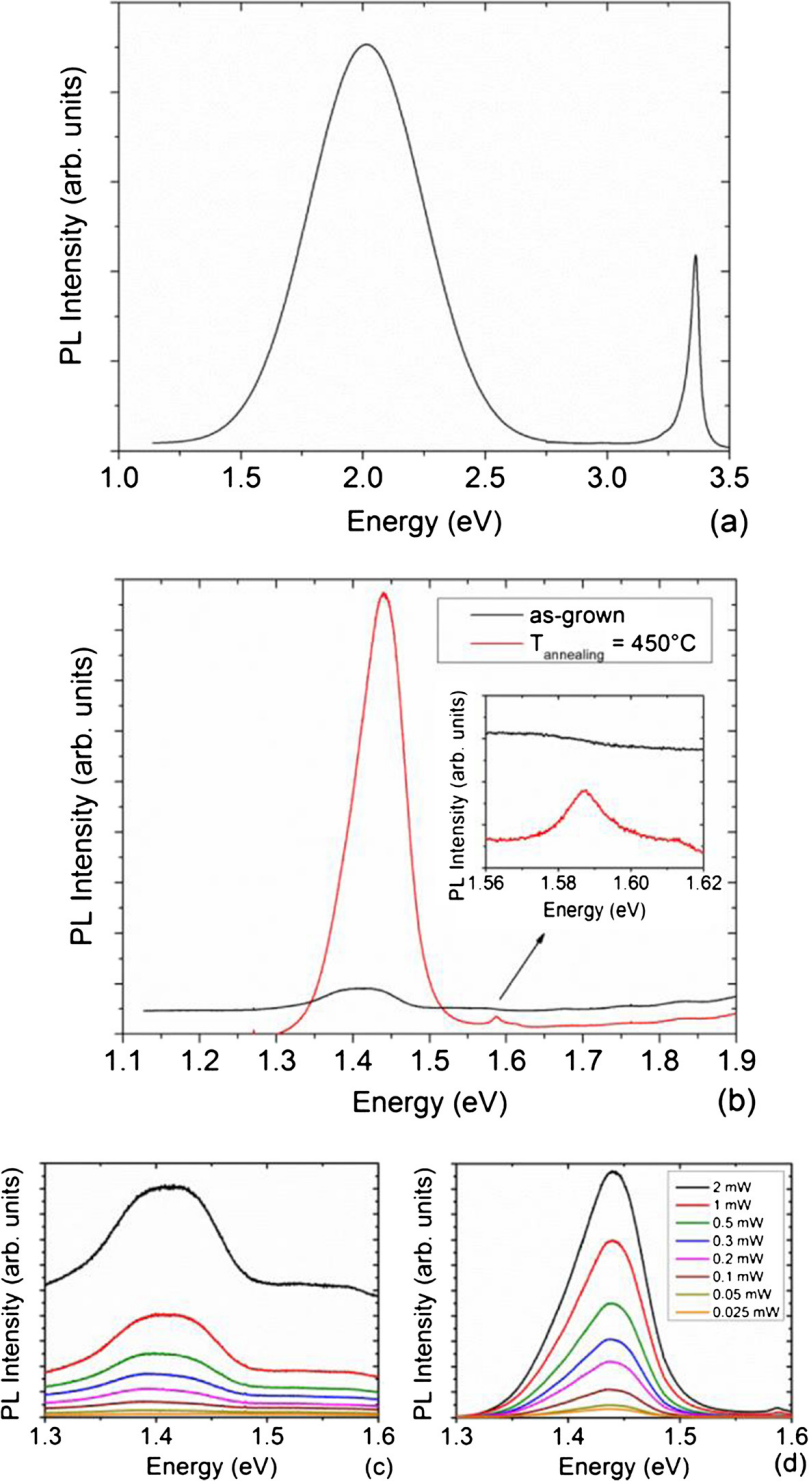
**Optical properties.** 5 K PL spectra of **(a)** bare ZnO NWs and **(b)** as-grown and annealed ZnO/CdTe core-shell NW arrays at 450°C for 1 h. The excitation power and beam size are 1 mW and 100 µm, respectively. Excitation power-dependent 5 K PL spectra of the **(c)** as-grown and **(d)** annealed ZnO/CdTe core-shell NW. arrays at 450°C for 1 h.

The 5 K PL spectra of the as-grown and annealed ZnO/CdTe core-shell NW arrays are presented in Figure 5 and divided into four distinct regions. The near-band edge (NBE) of the ZnO NWs is governed by radiative transitions of neutral donor bound excitons at 3.36 eV, as shown in Figure 5a [3,59]. The red-orange emission band occurs at about 2.0 eV in bare ZnO NWs and may be related to native point defects involving interstitial oxygen [3]. The deposition of the CdTe NGs on top of the ZnO NWs influences the PL spectra in the energy range of 1.8 to 2.5 eV. The NBE of the as-grown CdTe NGs does not exhibit any significant luminescence. Instead, a broad emission band centered at 1.41 eV arises, as revealed in Figure 5b. The dependence of the intensity of the broad emission band on the excitation power follows a power law [60] with a power coefficient of 0.7 ± 0.05, which is smaller than 1. This indicates that radiative transitions of donor acceptor pairs (DAP) are involved in the broad emission band. Basically, a wide number of impurities can substitute for tellurium (i.e., chlorine, bromine, and iodine) or cadmium (i.e., aluminum, gallium, and indium) and form the so-called ‘A-centers’ with cadmium vacancies in the nearest neighbor sites [61]. The chemical analysis of the CdTe powder by glow discharge mass spectrometry reveals that chlorine is the dominant impurity. Chlorine acts as a donor in CdTe by substituting for tellurium and leads to the formation of acceptor complexes [62]. The occurrence of chlorine donors and A-centers results in compensation processes. Chlorine A-centers contribute to the radiative transitions of DAPs in the broad emission band centered at 1.41 eV; the zero phonon line (ZPL) is located at the higher energy of 1.477 eV [63]. The strong coupling of chlorine A-centers with LO phonons results in a Huang-Rhys constant of about 2.2, leading to a higher intensity of the first and second LO phonon replica at 1.455 and 1.434 eV, respectively. Other contributions of aluminum and indium A-centers can also superimpose to the contribution of chlorine A-centers at lower energy since aluminum and indium have a significant residual concentration of several ppm [61]. More importantly, the PL spectra of the annealed ZnO/CdTe core-shell NWs are strongly affected by the CdCl_2_ heat treatment. The NBE of the annealed CdTe NGs arises at 1.589 eV, as shown in Figure 5b. Its dependence on the excitation power yields a power coefficient of 1.38 ± 0.1 (i.e., >1.2), showing that radiative transitions of bound excitons are involved [60]. The occurrence of excitonic type transitions indicates that the crystallinity of the CdTe NGs is strongly improved after CdCl_2_ heat treatment, which is in agreement with the previous structural analysis. Furthermore, the excitonic peak at 1.589 eV can be assigned with excitons bound to chlorine A-centers [61,62]. Correlatively, the intensity of the broad emission band centered at 1.44 eV is strongly increased after CdCl_2_ heat treatment, as already reported in CdTe thin films after HCF_2_Cl heat treatment [63], and its energy position is blueshift. A power coefficient of about 0.65 ± 0.05 is deduced from its dependence on the excitation power, pointing out that radiative transitions of DAPs are still involved [60]. The CdCl_2_ heat treatment favors the incorporation of chlorine atoms inside the CdTe NGs at the expense of other impurities as seen by the blueshift of the broad emission band. The role of chlorine is hence critical: first, chlorine forms A-centers by substituting for tellurium and linking with cadmium vacancies on the nearest neighbor sites; second, chlorine acts as an efficient passivating agent as deduced from density functional total-energy calculations [38]. Chlorine is thus able to passivate the dangling bonds of GBs, reducing the density of nonradiative recombination centers in their center [64] and enhancing the crystallinity of CdTe NGs.

### Effects on the photovoltaic properties of ZnO/CdTe core-shell NW arrays

**Figure 6:**
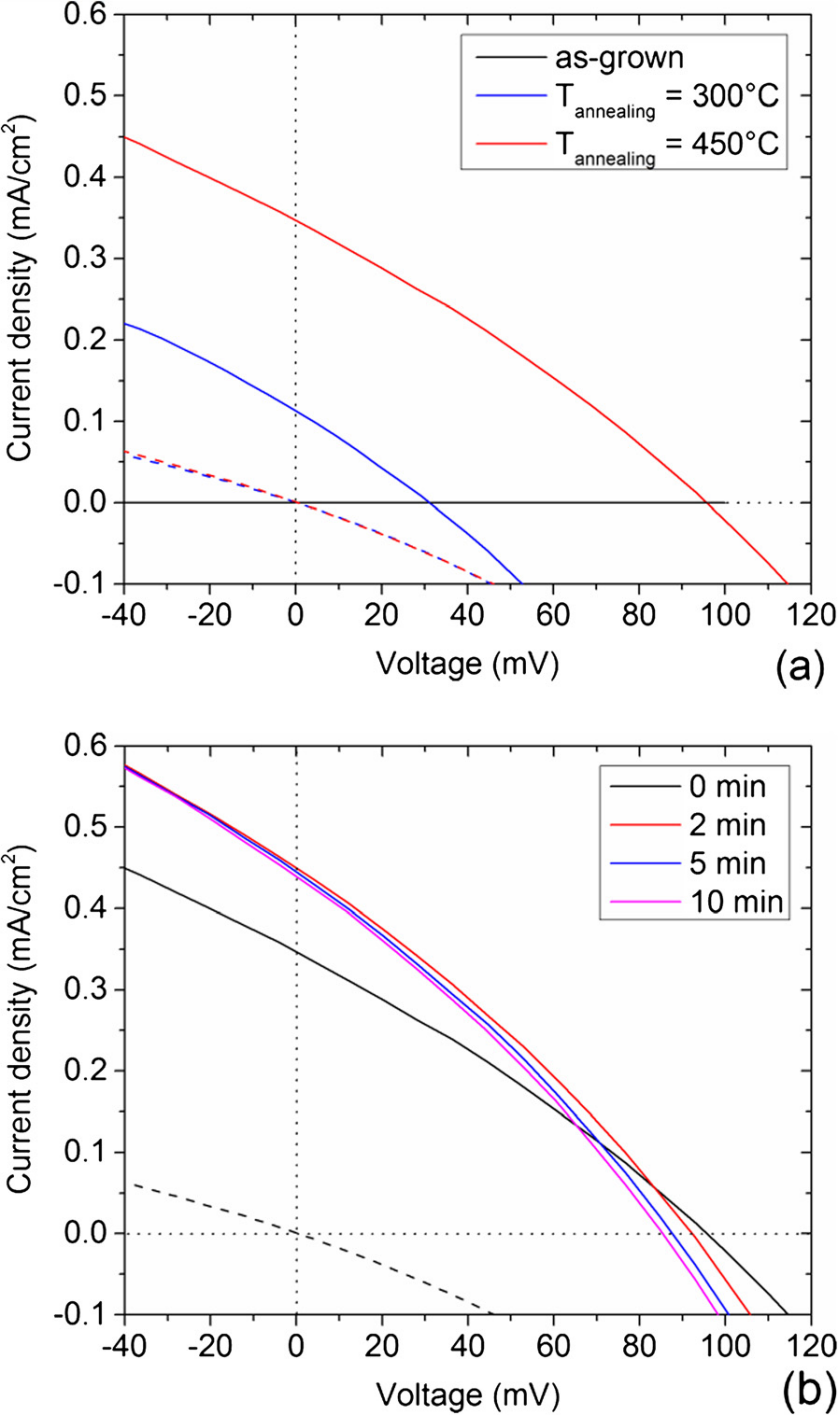
**Photovoltaic properties. (a)** *J*(*V*) characteristics of the as-grown and annealed ZnO/CdTe core-shell NW arrays at 300°C and 450°C for 1 h, under dark conditions (dashed lines) and AM 1.5G standard illumination conditions (solid lines). **(b)** *J*(*V*) characteristics of annealed ZnO/CdTe core-shell NW arrays at 450°C for 1 h under dark conditions (dashed line) and AM 1.5G standard illumination conditions (solid lines). The illumination is performed for a varying time (i.e., light-soaking effects).

**Figure 7:**
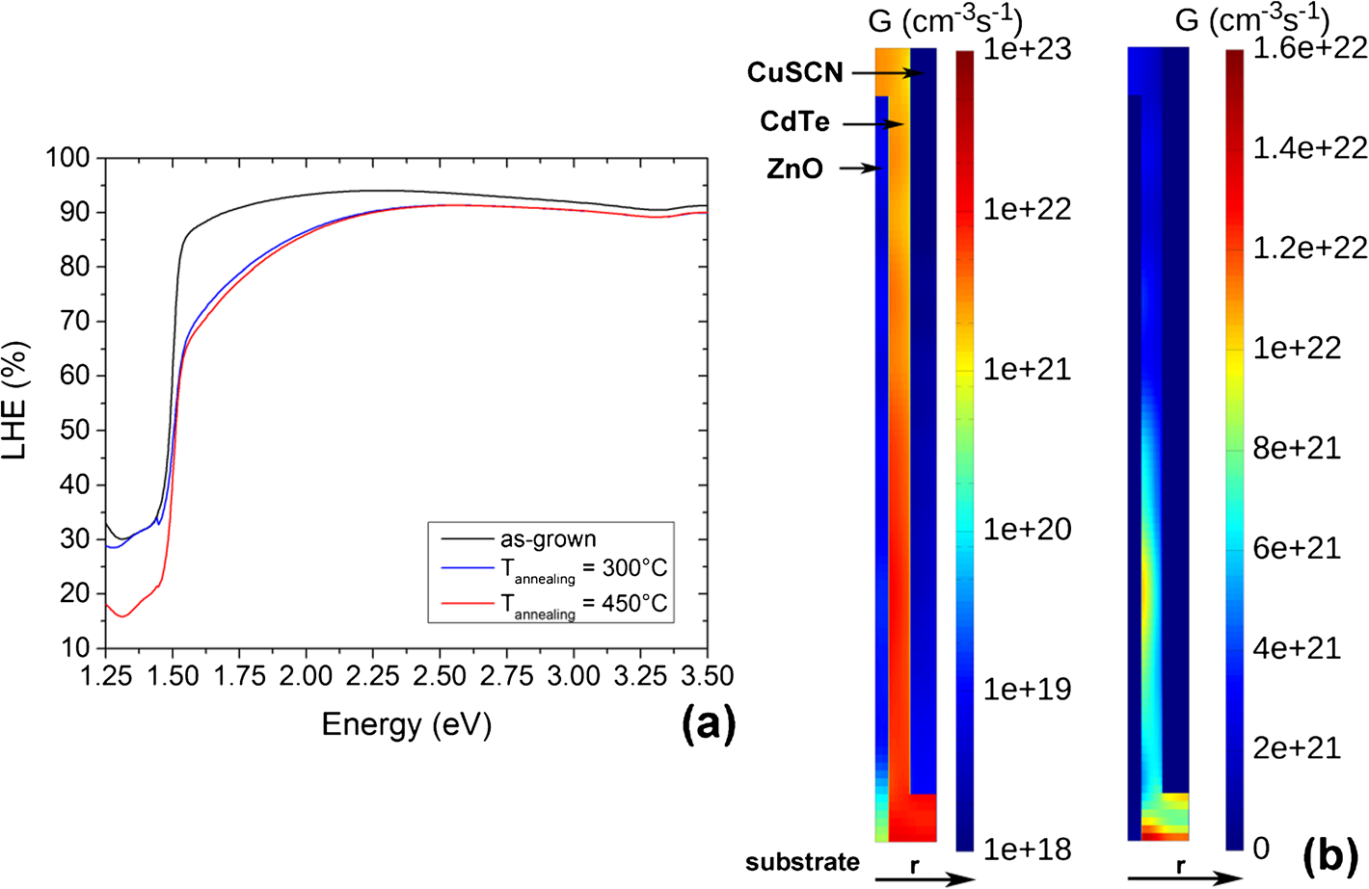
**Light-harvesting efficiency and polychromatic radial optical generation rate. (a)** Light-harvesting efficiency (LHE) of the as-grown and annealed ZnO/CdTe core-shell NW arrays at 300°C and 450°C for 1 h, respectively. **(b)** Maps of the polychromatic radial optical generation rate in ZnO/CdTe core-shell NW arrays plotted in both logarithmic (left) and linear (right) color scales. The optical simulations from RCWA are performed with the following stacking and geometrical dimensions: glass substrate (thickness = 1 mm), FTO thin films (thickness = 300 nm), ZnO seed layer (thickness = 20 nm), ZnO NWs (length = 1 μm, diameter = 75 nm, period = 345 nm, correlated spacing = 150 nm), CdTe shell (thickness = 60 nm), and CuSCN layer (thickness = 1 μm). The Au back-side contact is taken as semi-infinite.

**Figure 8:**
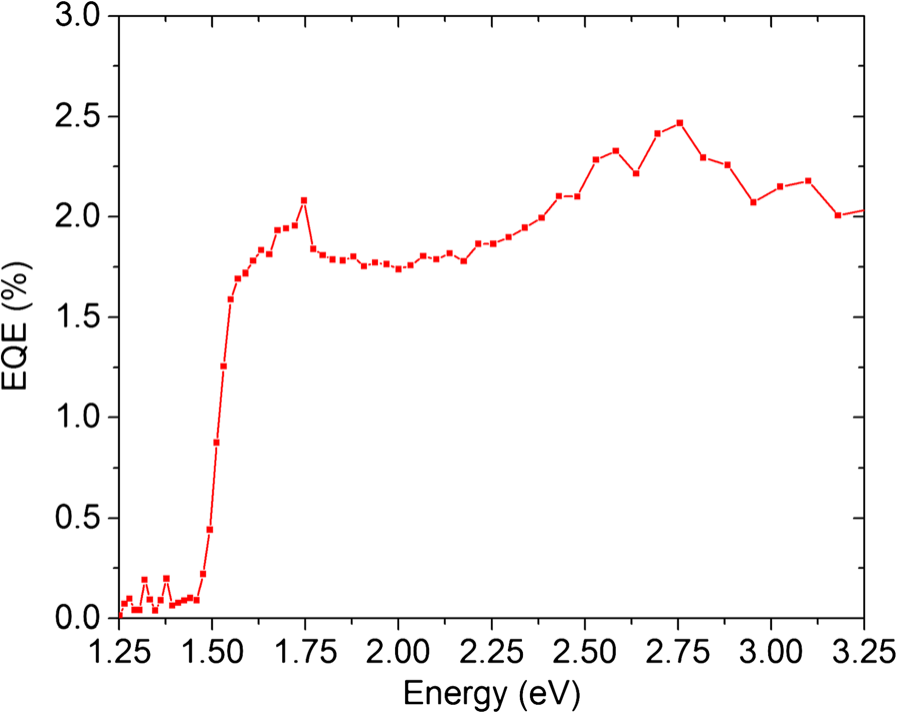
EQE measurements of the annealed ZnO/CdTe core-shell NW arrays at 450°C for 1 h.

The *J*-*V* characteristics under AM 1.5G standard illuminations, light-harvesting efficiency, and EQE measurements are presented in Figures 6, 7 and 8 for the ZnO/CdTe core-shell NW arrays. The main photovoltaic properties are given in Table 1. The as-grown ZnO/CdTe core-shell NW arrays only present a low photovoltaic effect with an open-circuit voltage (*V*_OC_) of 36 mV and a very poor short-circuit current density (*J*_SC_) of the order of several nA/cm^2^. Interestingly, the CdCl_2_ heat treatment is highly favorable for the photovoltaic properties of the annealed ZnO/CdTe core-shell NW arrays. As annealing temperature is raised from 300°C to 450°C, their photovoltaic properties are strongly enhanced, as shown in Figure 6a. A *V*_OC_ and *J*_SC_ of 96 mV and 0.35 mA/cm^2^, respectively, are generated in the ZnO/CdTe core-shell NW arrays annealed at 450°C. It is worth noticing that the CdCl_2_ heat treatment more strongly affects the *J*_SC_ than the *V*_OC_: the *V*_OC_ and *J*_SC_ are improved by a factor of 2.7 and 1.2 × 10^5^, respectively. In contrast, the filled factor (FF) does not seem to depend on post-growth heat treatment. The chlorine doping of CdTe NGs and the related GB passivation following the CdCl_2_ heat treatment are thus beneficial for the photovoltaic properties. The best photovoltaic properties only result in a photo-conversion efficiency of about 0.01%: this is fairly low as compared to the photo-conversion efficiency of 4.74% for ZnO/CdSe [65], 4.15% for ZnO/CdS/CdSe [66], and 4.17% for ZnO/In_2_S_3_/CuInS_2_ NW arrays [67]. However, it has widely been reported that the photovoltaic properties of ZnO/CdTe core-shell NW arrays are poor [22,24,25,27,29,32]. The low *V*_OC_ may originate from the occurrence of cracks in the CuSCN thick layer acting as the hole-collecting layer, which could also increase the series resistance [32]. In contrast, the *J*_SC_ depends, in addition to the incident spectral flux density, on the EQE, which is the number of collected charge carriers divided by the number of incident photons. The EQE for the annealed ZnO/CdTe core-shell NW arrays is about 2% above the bandgap energy of 1.5 eV for CdTe, as shown in Figure 8. Basically, the EQE is equal to the internal quantum efficiency (IQE) multiplied by the light-harvesting efficiency. Still, the light-harvesting efficiency is fairly high in ZnO/CdTe core-shell NW arrays, as revealed in Figure 7a: the light-harvesting efficiency is typically larger than 90% at the energy of 2.36 eV (i.e., the wavelength of 525 nm at the maximum of the solar irradiance). This is in agreement with the systematic optical simulations of the ideal *J*_SC_ by RCWA, which have emphasized the large absorption capability of ZnO/CdTe core-shell NW arrays [20]. As a consequence, the low *J*_SC_ and EQE arise from the poor IQE: this indicates that most of the photo-generated charge carriers in CdTe NGs is lost. The location where the charge carriers are photo-generated is given in Figure 7b, by the maps of the polychromatic radial optical generation rate. Interestingly, most of the charge carriers are actually photo-generated in the CdTe shell, owing to its bandgap energy of 1.5 eV in contrast to the wide bandgap energy of ZnO and CuSCN. A smaller proportion of the incident light is still absorbed in the ZnO NWs, especially for lower wavelength. More importantly, the optical generation rate is significantly decreased from the bottom to the top of the ZnO/CdTe core-shell NW arrays, as shown in Figure 7b. The vast majority of charge carriers is even photo-generated at the extreme bottom of the ZnO/CdTe core-shell NW arrays inside the CdTe shell. It is expected that the main critical point for these solar cells is related to the collection of the photo-generated charge carriers. The absence of structural relationship (i.e., hetero-epitaxy) in between the ZnO NWs and CdTe NGs, as previously shown by the HRTEM images in Figure 3, or the ZnO/CdTe band alignment may play a detrimental role on the electron transfer from the CdTe NGs into the ZnO NWs [68]. However, solar cells made from ZnO/CdTe epitaxy-free planar layers have already reached the photo-conversion efficiency of 12.3%, which clearly indicates that the combination of ZnO with CdTe can work for photovoltaic devices [18]. It is also worth noticing that dye-sensitized solar cells made from identical ZnO NWs can lead to the photo-conversion efficiency as high as 4.7%, which somehow points out that the electron conduction in ZnO NWs and collection from FTO top-side contact are not the limiting physical processes [11]. Instead, the poor collection of the holes from the CuSCN/Au back-side contact is presumably expected to be critical. The holes that are mainly photo-generated at the extreme bottom of the ZnO/CdTe core-shell NW arrays inside the CdTe shell just like the electrons are much farther from the Au back-side contact than the electrons from the FTO top-side contact. The poor collection of the holes may be due to (i) the low conductivity of the CuSCN layer and (ii) the CdTe/CuSCN band alignment. The diffusion of copper in the CdTe shell may occur as well, but the deposition of the CuSCN layer is achieved at the low growth temperature of 100°C. Eventually, light-soaking effects occur in the annealed ZnO/CdTe core-shell NW arrays, as revealed in Figure 6b. After 2 min of AM 1.5G standard illuminations, the *J*_SC_ increased from 0.35 to 0.45 mA/cm^2^ while slightly reducing the *V*_OC_. The relative decrease in the *V*_OC_ can be related to an increase in the solar cell temperature, which was not monitored. However, the increase in the *J*_SC_ is too high to be only due to solar cell temperature effects. Metastable effects in p-CdTe/n-CdS heterojunction solar cells or modules have already been reported, originating from copper diffusion from the back-side contact [69,70]. Here, light-soaking effects are more likely associated with the saturation of trap centers in CdTe NGs, leading to the increase in the *J*_SC_ through the collection of more electrons and holes [71].

**Table 1:** Photovoltaic properties of the resulting solar cells

**Solar cells**	***J***_**SC **_**(mA/cm**^**2**^**)**	***V***_**OC **_**(mV)**	**FF (%)**	***η *** **(%)**
As-grown	3 × 10^-6^	36	26.2	2.8 × 10^-8^
Annealed 300°C, 1 h	0.11	31	27.0	9.2 × 10^-4^
Annealed 450°C, 1 h	0.35	96	28.5	9.6 × 10^-3^
2 min	0.45	92.5	29.3	1.2 × 10^-2^
5 min	0.445	88	28.4	1.15 × 10^-2^
10 min	0.44	85.5	29.5	1.1 × 10^-2^

The solar cells are composed of as-grown and annealed ZnO/CdTe core-shell NW arrays covered with the CuSCN/Au back-side contact. The ZnO/CdTe core-shell NW arrays annealed at 450°C for 1 h are covered with the CuSCN/Au back-side contact and illuminated under AM 1.5G standard conditions for a varying time prior to the *J*(*V*) characteristic measurements.

## Conclusions

The effects of the CdCl_2_ heat treatment are investigated on the structural ordering, doping, and photovoltaic properties of ZnO/CdTe core-shell NW arrays grown by low-cost deposition techniques. It is found by FESEM images and XRD measurements that recrystallization phenomena are induced in CdTe NGs by the CdCl_2_ heat treatment. Their crystallinity is improved through the formation of well-defined facets and GBs while grain growth and texture randomization occur. The initial texture of the as-grown CdTe NGs along the <531 > direction is driven by strain energy minimization and is slightly reduced in favor of the <100 > orientation after the CdCl_2_ heat treatment. The occurrence of a crystalline tellurium phase is revealed by Raman scattering measurements and strongly enhanced after the CdCl_2_ heat treatment. The crystalline tellurium phase may decorate GBs in CdTe NGs. Furthermore, the chlorine doping of CdTe NGs is achieved after the CdCl_2_ heat treatment. The formation of chlorine A-centers is shown by PL measurements; after the CdCl_2_ heat treatment, radiative transition of excitons bound to chlorine A-centers arise at 1.589 eV, while the intensity of the related emission band involving donor acceptor pairs at 1.44 eV is increased. It is also expected that chlorine can passivate GBs. The chlorine doping and passivation are beneficial for the photovoltaic properties of ZnO/CdTe core-shell NW arrays. The absorption properties of the as-grown and annealed ZnO/CdTe core-shell NW arrays are highly efficient, and about 80% of the incident light is absorbed in the spectral range of the solar irradiance. Most of the charge carriers are photo-generated at the bottom of the ZnO/CdTe core-shell NW arrays inside the CdTe shell, as shown by the maps of the optical generation rate computed from RCWA. The photovoltaic properties of the ZnO/CdTe core-shell NW arrays when covered with the CuSCN/Au back-side contact are strongly improved after the CdCl_2_ heat treatment but remain low. It is expected that the main limitation originates from the poor collection of the holes generated in the CdTe shell from the CuSCN/Au back-side contact. Eventually, the CdCl_2_ heat treatment should systematically be achieved for the fabrication of solar cells made from ZnO/CdTe core-shell NW arrays.

## Competing interests

The authors declare that they have no competing interests.

## Authors' contributions

VC, JG and EA carried out the fabrication of the ZnO NWs on top of ZnO seed layer and FTO/glass substrate. VC and SR achieved the deposition of the CdTe NGs with heat treatment, while JG made the deposition of the CuSCN/Au back-side contact. EA collected the SEM images, while PG and LR performed the XRD and TEM characterizations, respectively. LA and VC collected the Raman and PL spectra, respectively. VC performed the absorption measurements. JM achieved the optical simulations. JG and AKC performed the photovoltaic measurements of the solar cells. VC drafted the manuscript. All authors discussed the results and contributed to the final manuscript. All authors read and approved the final manuscript.
